# Decoding the sugar–strigolactone crosstalk: new frontier in plant growth and stress resilience

**DOI:** 10.1093/hr/uhaf278

**Published:** 2025-11-19

**Authors:** Yuhui Wang, Léo Gouaille, Jing Meng, Michael Nicolas, Laurent Ogé, Zhengrong Jiang, Laurent Crespel, Yanfeng Ding, José Le Gourrierec, Ganghua Li, Philippe Grappin, Soulaiman Sakr

**Affiliations:** Institut Agro, University of Angers INRAE, IRHS, SFR QUASAV, F-49000 Angers, France; College of Agronomy, Nanjing Agricultural University, Nanjing 210095, China; Institut Agro, University of Angers INRAE, IRHS, SFR QUASAV, F-49000 Angers, France; Institut Agro, University of Angers INRAE, IRHS, SFR QUASAV, F-49000 Angers, France; College of Landscape and Horticulture, Yunnan Agricultural University, Kunming 650201, China; Plant Molecular Genetics Department, Centro Nacional de Biotecnología-CSIC, Campus Universidad Autónoma de Madrid, Madrid, Spain; Institut Agro, University of Angers INRAE, IRHS, SFR QUASAV, F-49000 Angers, France; Institut Agro, University of Angers INRAE, IRHS, SFR QUASAV, F-49000 Angers, France; College of Agronomy, Nanjing Agricultural University, Nanjing 210095, China; Institut Agro, University of Angers INRAE, IRHS, SFR QUASAV, F-49000 Angers, France; College of Agronomy, Nanjing Agricultural University, Nanjing 210095, China; Institut Agro, University of Angers INRAE, IRHS, SFR QUASAV, F-49000 Angers, France; College of Agronomy, Nanjing Agricultural University, Nanjing 210095, China; Institut Agro, University of Angers INRAE, IRHS, SFR QUASAV, F-49000 Angers, France; Institut Agro, University of Angers INRAE, IRHS, SFR QUASAV, F-49000 Angers, France

## Abstract

Plants continuously integrate metabolic and hormonal signals to coordinate growth, development, and responses to environmental stimuli. Among these signals, sugars and strigolactones (SLs) have emerged as central regulators. Beyond serving as metabolic fuels, sugars act as signaling molecules that govern key developmental transitions and stress responses. SLs, a relatively recent addition to the phytohormone family, play pivotal roles in shaping plant architecture, modulating resource allocation, and facilitating environmental adaptation. While the individual signaling functions of sugars and SLs are well documented, their crosstalk remains an emerging and largely underexplored area of plant biology. This review synthesizes current knowledge on both the independent and interactive roles of sugar and SL signaling across critical developmental processes, including seed germination, hypocotyl elongation, root and shoot architecture, flowering, senescence, and plant responses to abiotic and biotic stress. By analyzing antagonistic and synergistic interactions, we point out several potential integrative hubs where metabolic and hormonal signals converge to fine-tune the final decision. Notably, the nodal roles of *BRC1/TB1* (*BRANCHED1/TEOSINTE BRANCHED1*), *FT* (*FLOWERING LOCUS T*), in mediating sugar–SL crosstalk in shoot branching, flowering, respectively, are highlighted. We also explore how sugar-SL interplay influences seed germination and plant adaptation to environmental stresses through shared regulators such as TOR (Target of Rapamycin) kinase, SnRK1 (Sucrose non-fermenting-1 Related Kinase 1), and SMXLs (Suppressor of MAX2-Like proteins). Understanding these interactions not only deepens our knowledge of fundamental plant biology but also offers new insights for improving the performance and resilience of crop and horticultural species.

## Introduction

As sessile organisms, plants have evolved sophisticated signaling systems to integrate internal metabolic cues with external environmental conditions. Nutrient and hormonal signaling represent one of the most ancient and evolutionarily conserved regulatory networks. Sugars are unique in that they act as both primary metabolites and signaling molecules, orchestrating developmental transitions from germination to senescence. Over the past few decades, extensive research has shed light on the complex nature of sugar signaling pathways. These pathways involve complex networks that interact extensively with phytohormones such as auxins, gibberellins (GAs), cytokinins (CKs), abscisic acid (ABA), ethylene (ET), jasmonic acid (JA), and salicylic acid (SA). These interactions coordinate cell proliferation, energy homeostasis, and stress responses [1–5].

Despite substantial progress in elucidating sugar–hormone crosstalk, the interaction between sugars and strigolactones (SLs; a relatively recent and distinctive class of carotenoid-derived phytohormones) signaling pathways remains underexplored. SLs play pivotal roles in regulating key aspects of plant development and the establishment of symbiotic relationships with arbuscular mycorrhizal fungi and rhizobia [6–8]. Moreover, SLs are increasingly recognized as essential mediators of plant responses to various abiotic and biotic stresses, including drought, nutrient limitation, and pathogen attack [9–11].

Given the central role of sugars in both energy homeostasis and signaling, their interaction with SLs represents a compelling regulatory nexus in plant development and stress responses. Emerging studies indicate that sugars can influence SL biosynthesis, signaling sensitivity, and downstream transcriptional responses [12]. Conversely, SLs may modulate sugar metabolism, transport, and signaling. Understanding how these two systems integrate is essential to unravelling the coordination of plant growth, development, and resilience. These regulatory interactions are particularly important for horticultural crops, as they are often cultivated using intensive management systems and are increasingly challenged by abiotic and biotic pressures. This review summarizes the latest research on sugar and SL signaling pathways and their points of convergence. We examine their interactions across pivotal developmental stages—including germination, hypocotyl elongation, root and shoot architecture, flowering, and senescence —as well as their functions in responses to abiotic and biotic stresses. By highlighting mechanistic models and unresolved questions, we aim to provide a conceptual framework for future research on the interaction between sugars and SLs in plant biology, including horticultural species.

## Overview of sugar and SL signaling: orchestrators of plant development and stress resilience

### Sugar sensing and signaling pathways

**Figure 1:**
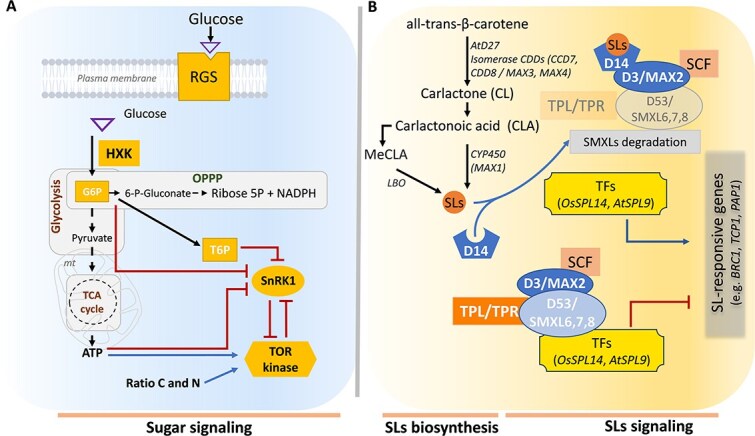
Overview of the metabolism and signaling of sugars and SLs. **A.** Sugar metabolism and signaling in plants. Glucose is phosphorylated by hexokinase (HXK) in the cytoplasm to form glucose-6-phosphate (G6P), a key intermediate that feeds into both glycolysis/the tricarboxylic acid (TCA) cycle and the oxidative pentose phosphate pathway (OPPP). In addition to its enzymatic role, HXK also acts as a primary glucose sensor, linking sugar metabolism to signaling pathways. Sugar sensing is mediated by HXK and its associated downstream metabolic processes, including glycolysis, the TCA cycle, and the OPPP. Trehalose-6-phosphate (T6P) serves as a proxy for sucrose availability and plays a central role in aligning plant developmental processes with carbohydrate status. Two major energy-sensing kinases function downstream of sugar perception: Sucrose non-fermenting Related Kinase 1 (SnRK1), which is activated under energy-deficient conditions, and the Target of Rapamycin (TOR) kinase, which integrates energy and nutrient cues, particularly the carbon-to-nitrogen (C/N) ratio. SnRK1 and TOR kinase antagonistically regulate each other. Sugar perception can also occur at the plasma membrane independently of HXK, via the Regulator of G-protein Signaling 1 (RGS1), which detects extracellular (apoplastic) glucose. Blue arrows represent activation, red blunt-ended lines indicate repression, and dark dotted lines denote metabolic pathways. **B.** SLs metabolism and signaling. All-trans-β-carotene is converted into carlactone (CL) through a series of enzymatic steps involving the carotenoid isomerase DWARF27 (D27), and the carotenoid cleavage dioxygenases CCD7 (also known as MORE AXILLARY GROWTH3, MAX3) and CCD8 (MAX4). CL is subsequently oxidized to carlactonoic acid (CLA), a key intermediate in SL biosynthesis. The cytochrome P450 enzyme MORE AXILLARY GROWTH1 (MAX1) and the oxidoreductase LATERAL BRANCHING OXIDOREDUCTASE (LBO) further catalyze the production of canonical and non-canonical SLs. SLs are perceived by the receptor DWARF14 (D14), an α/β hydrolase. In the absence of SLs, transcriptional repressors SUPPRESSOR OF MAX2 1-LIKE 6, 7, and 8 (SMXL6, SMXL7, and SMXL8) interact with the co-repressors TOPLESS/TOPLESS-RELATED (TPL/TPR) to inhibit the expression of SL-responsive genes, including *BRANCHED1* (*BRC1*), *TEOSINTE BRANCHED1/CYCLOIDEA/PCF1* (*TCP1*), and *PRODUCTION OF ANTHOCYANIN PIGMENT1* (*PAP1*). Upon SL perception, D14 forms a complex with the F-box protein MORE AXILLARY GROWTH2 (MAX2), also known as DWARF3 (D3) in rice. This SCF (SKP1-CULLIN-F-box) E3 ubiquitin ligase complex targets SMXL6/7/8 proteins for ubiquitination and degradation via the 26S proteasome. This degradation relieves transcriptional repression and enables the activation of SL-responsive gene expression. Blue arrows represent activation, red blunt-ended lines indicate repression, and dark dotted lines denote metabolic pathways.

Sugars, primarily sucrose and glucose, serve as metabolic fuels and central signaling molecules that regulate development and stress responses in plants [2, 4, 13], including horticultural species [14]. Multiple sugar sensors and energy signaling pathways contribute to this regulation (Fig. 1A). Hexokinase 1 (HXK1) is an evolutionarily conserved glucose kinase found in most living organisms. It acts as both a glucose sensor and an enzyme [15, 16], modulates photosynthesis, leaf formation, seedling growth, senescence, shoot branching, fruit development, secondary metabolites (e.g. anthocyanin and flavonoid synthesis), and stress resilience [17–24]. HXK1 influences transcriptional networks that integrate sugar, light, and hormone signals in Arabidopsis [25]. Through its catalytic conversion of glucose into glucose-6-phosphate (G6P), HXK fuels both the glycolytic/tricarboxylic acid (TCA) cycle and the oxidative pentose phosphate pathway (OPPP) that respectively provide energy metabolism and reducing power (NADPH, Nicotinamide adenine dinucleotide phosphate) essential for plant growth and development [26–28] (Fig. 1A). The derived product of G6P, the trehalose-6-phosphate (T6P), reflects sucrose status [29], and also regulates various developmental processes and stress responses in multiple species such as Arabidopsis, tomato, and grapevine [30–35]. Generally, this control is achieved through the inhibition of SnRK1 (Sucrose non-fermenting-1-related protein kinase 1), a kinase that reprograms metabolism under low-energy conditions (Fig. 1A). SnRK1 represses growth and activates catabolic processes and stress defences in response to sugar starvation [36]. It influences the activity of transcription factors such as bZIPs and modulates germination, flowering, and senescence [23, 36, 37]. In contrast, TOR (Target of Rapamycin) kinase, which belongs to the phosphatidylinositol 3-kinase-related kinase (PIKK) family of atypical serine/threonine protein kinases [38], promotes anabolic growth processes under energy-rich conditions [39]. TOR supports protein synthesis, root growth, and nutrient assimilation, while repressing autophagy and senescence [23, 40]. TOR and SnRK1 kinases are generally considered to antagonize each other in plants (Fig. 1A). Finally, AtRGS1 (Regulator of G-protein signaling 1) functions as a membrane receptor or co-receptor for D-glucose and mediates HXK-independent glucose sensing [41, 42]. It modulates various processes such as growth, development, stress tolerance [42–44] and response to environmental variation, as shown in tomato [45, 46]. Together, these pathways enable plants to adjust their growth and development dynamically in response to carbon availability.

### SL biosynthesis and signaling pathways

SLs are terpenoid lactones derived from carotenoids. They were initially identified for their role in stimulating the germination of parasitic plants [8, 47, 48] and more than 20 natural SLs have been identified [49, 50]. SLs were later identified as key regulators of numerous physiological and developmental processes in diverse plant species—including Arabidopsis, rice, tomato, petunia, grapevine—such as seed germination, mineral uptake, root and shoot architecture, leaf senescence, anthocyanin accumulation, fruit development and quality, as well as response to abiotic and biotic stresses [51–55]. SLs also contribute to establishing beneficial symbioses with mycorrhizal fungi and enhancing plant nutrient acquisition [12, 56]. SL biosynthesis begins with all-trans-β-carotene and proceeds through enzymatic steps involving D27 (DWARF27), CCD7 (carotenoid cleavage dioxygenase 7), CCD8, and cytochrome P450 enzyme (MORE AXILLARY GROWTH1, MAX1) producing carlactone and its derivatives (e.g. carlactonoic acid) (Fig. 1B). In Arabidopsis, LATERAL BRANCHING OXIDOREDUCTASE (LBO) functions downstream of MAX1, producing non-canonical SLs [57]. Xu et al. [58] identified CARBOXYLESTERASE 15 (CXE15), an α/β-hydrolase enzyme, as involved in SL catabolism (Fig. 1B). These reactions occur in both roots and shoots and are tightly regulated by developmental and environmental cues.

The perception and signaling of SLs are mediated by the SL receptor DWARF14 (D14), an α/β-hydrolase [59] (Fig. 1B). Upon SL binding, D14 interacts with the F-box protein MAX2 (MORE AXILLARY GROWTH2) (or D3 (DWARF3) in rice), forming the SCF-MAX2 complex. This complex targets the SL-signaling repressors SMXLs (Suppressor of Max1-like proteins) (SMXL6, SMXL7, SMXL8 in Arabidopsis; DWARF53 (D53) in rice; PsSMXL7 in pea), which are interactors of the transcriptional corepressors TOPLESS (TPL) and TPL-RELATED (TPR), for degradation via the 26S proteasome pathway [60–64]. This proteasome-mediated turnover releases transcriptional repression of SL-dependent genes. SL signaling controls shoot branching, root architecture, senescence, and stress responses by regulating the expression of the key transcription factors (e.g. *BRC1*/*TB1*, *TCP1*, *SPLs*) [60, 63]. SLs also contribute to establishing beneficial symbioses with mycorrhizal fungi and enhancing plant nutrient acquisition [65]. Finally, SLs act in interaction with other phytohormones—such as auxin, ABA, CKs, and ET—as part of complex regulatory networks [66], as reported in tomato [67].

## Breaking dormancy: coordinated sugar and SL signaling in seed germination

**Figure 2:**
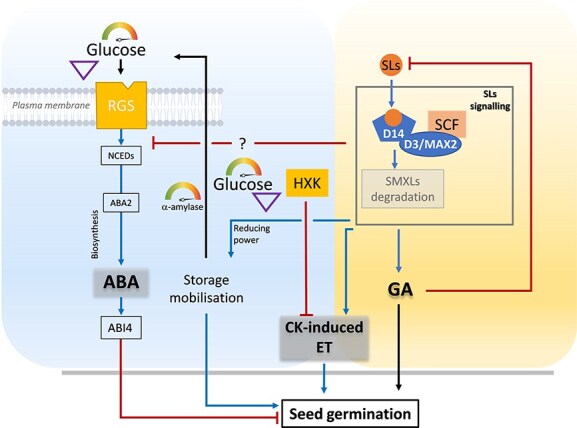
Negative crosstalk between sugar and SL signaling pathways in the regulation of seed germination. High glucose levels promote abscisic acid (ABA) biosynthesis through the activation of enzymes such as *NCEDs* (*9-cis Epoxycarotenoid Dioxygenase*) and *the short-chain alcohol dehydrogenase ABA2* (*Acid abscisic Deficient2*) reinforcing ABI4 (ABA-Insensitive 4)-mediated repression of seed germination. Glucose is sensed through two distinct pathways: the hexokinase (HXK)-dependent pathway, which suppresses cytokinin (CK)-induced ethylene (ET) biosynthesis, and a HXK-independent pathway involving Regulator of G-protein Signaling 1 (RGS1), which promotes ABA synthesis and signaling. In contrast, SLs stimulate seed germination by activating the D14-MAX2-SCF complex, which targets SUPPRESSOR OF MORE AXILLARY GROWTH2-LIKE6, 7 and 8 (SMXLs) ) for degradation. This promotes CK-induced ET biosynthesis and gibberellin (GA) signaling, both of which enhance germination and hypocotyl elongation. In this context, ABA, CK, and ET may act as key converging nodes integrating sugar and SL signaling pathways to finely tune the timing and efficiency of seed germination. The specific identity of the SMXLs involved remains to be clarified. Blue arrows represent activation, red blunt-ended lines indicate repression.

Seed germination is critical for agricultural yield and production as it directly determines seedling establishment, subsequent plant growth, and overall crop productivity [68]. This developmental transition is regulated by a finely tuned interplay of endogenous phytohormones [69, 70] and environmental factors [71]. Key hormonal regulators include ABA that enforces dormancy and inhibits germination and GAs that promote germination and early seedling growth. This hormonal balance is modulated by metabolic status, with sugars and SLs playing pivotal, context-dependent roles. During germination, sugars serve a dual function: they act as metabolic fuels—primarily mobilized from starch reserves—and as signaling molecules that influence hormonal pathways [72, 73]. In Arabidopsis, sugars support germination by providing energy for cellular metabolism. However, glucose excess can inhibit germination by enhancing ABA biosynthesis and signaling, which in turn suppresses CK biosynthesis and signaling genes, primarily through the action of HXK1 and AtRGS1 (Fig. 2) [72–76]. In this context, sugars upregulate ABA biosynthetic genes such as *NCED3* (*9 cis-Epoxycarotenoid Dioxygenase 3*) and *the short-chain alcohol dehydrogenase ABA2* (*Acid Abscisic Deficient 2*), as well as ABA-responsive transcription factors like *ABI4* (*ABA-Insensitive 4*) [46, 77, 78]. Depending on the sugar concentration and the developmental stage, the interaction between sugar and ET/CK can either enhance or reduce ABA signaling [79, 80]. These interactions ensure that germination is promoted only under favorable internal and external conditions, and that sugar homeostasis is maintained to coordinate energy metabolism and hormone responses. Initially identified as germination stimulants for parasitic plants such as Striga and Orobanche [8, 59], SLs have also been recognized as important regulators of seed germination in non-parasitic plants, particularly under abiotic stress conditions [54, 81, 82]. SLs critically regulate seed germination in horticultural species. GR24, a synthetic analog of SLs, alleviates salt stress and stimulates seed germination in cucumber by enhancing antioxidant capacity [82] and boosts tomato germination by promoting starch breakdown through the activation of α-amylase activity [83]. Similar effects have been reported in tomato and Arabidopsis, where SLs help alleviate salinity and heat stress during germination [81, 83]. SLs promote germination by modulating the hormonal balance, specifically by reducing ABA levels and enhancing GA accumulation, thereby decreasing the ABA/GA ratio [84]. This hormonal shift facilitates dormancy release and radicle emergence, even under adverse environmental conditions. SLs induce CK production in parasitic plants, and hypothetically also in non-parasitic plants, to further promote seed dormancy release [84–86]. Additionally, SLs have been implicated in crosstalk with ET signaling: in Striga, SLs induce ET biosynthesis via CK biosynthesis, which subsequently triggers germination [84, 87]. Overall, seed germination is orchestrated by an intricate network of sugar metabolism, hormone signaling (including SLs), and environmental inputs. Sugar and SL interplay, especially in ABA and ET/CK pathways, is central in determining the timing and success of germination. Modulating sugar and SL signaling offers opportunities to improve seed germination quality in horticultural species with significant economic importance. However, these effects are highly context-dependent, varying with species, developmental stage, and environmental conditions. Further research is essential to unravel the molecular mechanisms underlying this dynamic regulatory network.

## Early growth decisions: sugar and SL antagonism in hypocotyl elongation

Hypocotyl elongation enables the emerging seedling to reach light sources and initiate autotrophic growth, both of which are crucial for successful establishment and subsequent development in crop and horticultural species. Hypocotyl elongation is tightly regulated by carbon availability and hormonal signaling. In soybean and melon seedlings, hypocotyl elongation is modulated by light quality, affecting sugar metabolism [88]. Sugars themselves play a dual role in promoting hypocotyl elongation, serving as both energy sources and signaling molecules. Key sugar signaling components—T6P, HXK1, and SnRK1—mediate these effects. In Arabidopsis, T6P enhances elongation by promoting auxin biosynthesis, primarily through activation of phytochrome interacting factors (PIFs), which play a central role in growth responses in darkness [89, 90]. In contrast, SnRK1 acts antagonistically by repressing growth under low-sugar or stress conditions [90]. HXK1, particularly in guard cells, integrates sugar and light signals and contributes to PIF4 activation, thereby promoting hypocotyl elongation under the appropriate conditions [91]. Moreover, the TOR kinase pathway links sucrose availability to hormonal growth signals, including BR (Brassinosteroid) and GAs, especially under dark or shaded conditions [92, 93]. In contrast to the growth-promoting role of sugars, SLs generally inhibit hypocotyl elongation, particularly under light conditions in Arabidopsis [94]. This inhibitory effect is mediated through the D14–MAX2 signaling module, which targets SMXL2—a positive regulator of elongation—for proteasomal degradation [63]. The degradation of SMXL2 leads to the upregulation of transcriptional repressors such as *DLK2 (DWARF14-LIKE2)* and *KUF1 (KARRIKIN UP-REGULATED F-BOX)*. These repressors then suppress hypocotyl growth [63]. The integration of sugar and SL signaling in regulating hypocotyl elongation remains poorly understood, and whether SMXL2 functions as a molecular hub for antagonistic sugar–SL crosstalk warrants further investigation.

## Finding the right balance between growth and restraint: integration of sugar and SL signaling in root architecture

**Figure 3:**
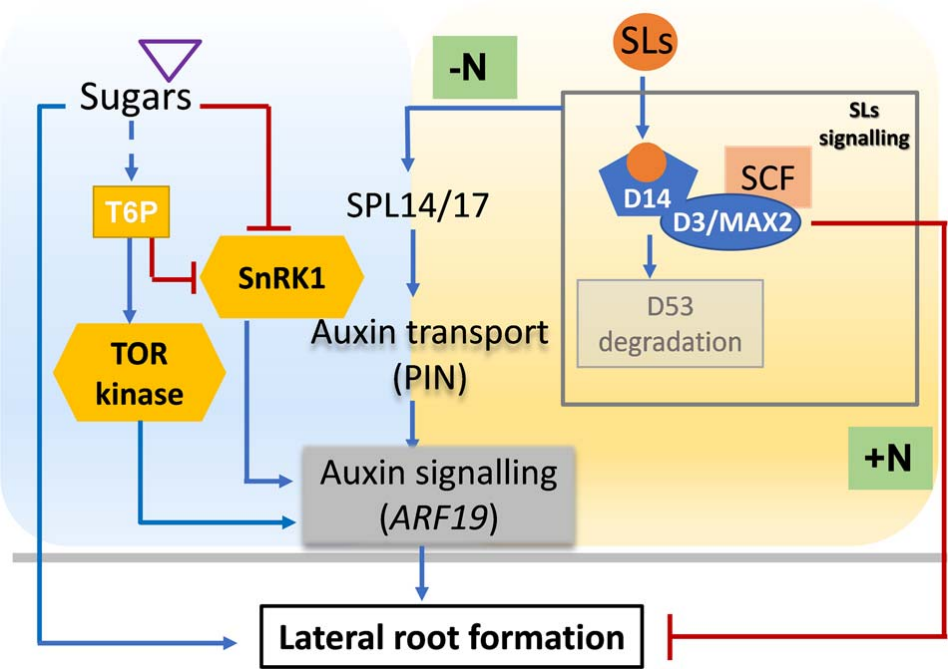
Proposed signaling network integrating sugar and SL pathways in the regulation of LR formation. Sugars promote LR formation, with trehalose-6-phosphate (T6P) acting upstream of the energy sensors Sucrose non-fermenting-1-Related Protein Kinase 1 (SnRK1) and Target of Rapamycin (TOR) kinase. These energy signaling pathways ultimately converge on auxin signaling, particularly through *Auxin Response Factor 19* (*ARF19*). Under favorable nutrient conditions, SLs repress LR development via a MORE AXILLARY GROWTH2 (MAX2)-dependent signaling pathway. In contrast, under nitrogen starvation, SLs promote LR formation by activating a regulatory cascade involving *SQUAMOSA PROMOTER BINDING PROTEIN-LIKE* (*SPL*) transcription factors, PIN-FORMED (PIN) auxin transporters, and *ARF19*. *ARF19* thus emerges as a key integrative node, modulated by both sugar availability and SL signaling. Blue arrows represent activation, while red blunt-ended lines indicate repression. +N, nitrogen-rich condition; -N, nitrogen limitation.

Root system architecture (RSA) plays a critical role in determining how effectively a plant acquires water and nutrients from the soil, particularly in horticultural species where root traits are linked to crop productivity, plant vigor and stress tolerance [95, 96]. RSA is primarily shaped by two key processes: primary root (PR) elongation and lateral root (LR) formation. It is dynamically regulated by the interplay between sugars and hormones, particularly auxin, in response to environmental signals [97–99]. Sugars serve as central regulators of LR formation through a complex mechanism involving T6P, and the core energy-signaling pathways: SnRK1 and TOR kinase (Fig. 3). In peach, SnRK1 is activated under moderate sucrose conditions such as low-energy or darkness and promotes root development by enhancing auxin signaling [98]. This occurs through the upregulation of *Indole-3-Acetic Acid Inducible 12* (*IAA12*) and *PIN-LIKES6* (*PILS6*) [98] and in Arabidopsis, via the activation of AUXIN RESPONSE FACTOR 19 (ARF19) by phosphorylating BASIC LEUCINE ZIPPER 63 (bZIP63) [100]. However, the TOR kinase acts as a sugar-sensitive checkpoint, permitting auxin-induced LR initiation only when sufficient sugar is present in the pericycle, where LRs are formed [99]. In Arabidopsis, TOR facilitates the translation of *ARF7*, *ARF19*, and *LBD16* (*LATERAL ORGAN BOUNDARIES DOMAIN 16*), which are key transcriptional regulators forming a core module in auxin-mediated LR formation. Additionally, TOR drives root meristem activity by promoting the glucose-induced phosphorylation of E2Fa, a transcription factor responsible for cell cycle activation [101]. The TOR pathway is also involved in the formation of adventitious roots in Arabidopsis and potato [102]. Importantly, T6P signal serves as a central regulator of energy status, linking sugar availability to LR development. Elevated T6P levels stimulate LR formation by activating TOR and simultaneously inhibiting SnRK1 (Fig. 3) [103]. T6P levels are transcriptionally modulated by auxin through repression of the T6P-degrading enzyme *TPPB* (*Trehalose-6-Phosphate Phosphatase B*), indicating a feedback loop in which auxin indirectly promotes its own downstream effects by raising T6P levels [103]. Thus, T6P acts as a critical integrator of sugar and hormonal signaling, mediating a metabolic checkpoint that allows LR formation only under favorable energy conditions. Conversely, SLs exert an opposing influence on RSA, generally promoting PR elongation while suppressing LR formation [104–107]. Under nutrient-rich conditions, SLs inhibit LR formation in Arabidopsis and pea through the MAX2-dependent pathway, which regulates auxin receptor (Transport Inhibitor Response 1; TIR1) and PIN-formed (PIN) auxin transporters [107, 108]. Consistently, SL-deficient or -insensitive mutants show increased LR density, whereas GR24 suppresses LR initiation through MAX2. Downstream components such as D53 and SMXL6/7/8 further modulate auxin transport and transcription [109]. Under nitrogen limitation, SL-induced degradation of D53 activates *SPL14* (*SQUAMOSA PROMOTER BINDING PROTEIN-LIKE14*) and its homolog *SPL17*, upregulating *OsPIN1b* and promoting seminal root elongation in rice [110]. Only a couple of lines of evidence suggest crosstalk between SLs and sugars in root development. In Arabidopsis, GR24 regulates root-hair length only in the presence of sucrose [111]. Similarly, in tomato and wheat roots, SLs reshape the metabolome, including sugar-associated pathways [112]. Together, sugar and SL signaling appear to integrate developmental programs with environmental and metabolic cues to optimize RSA. This integration may involve the coordinated regulation of auxin signaling and transport through SMXLs, TOR, and SnRK1, although the precise mechanisms and context-dependent interactions are still unclear.

## Bud fate under antagonistic control: sugar promotion and SL suppression of shoot outgrowth and bud dormancy

**Figure 4:**
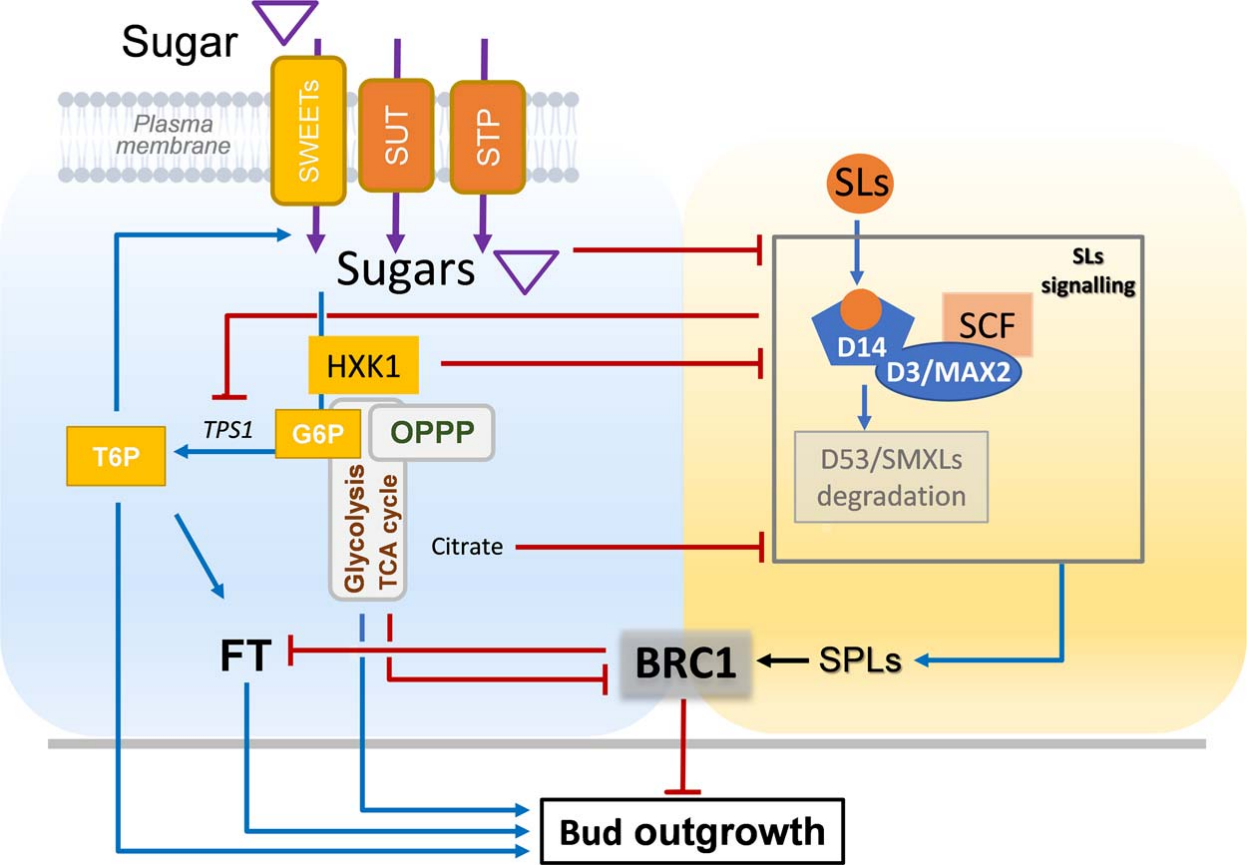
Sugar–SLs crosstalk in bud outgrowth regulation. Sugars promote bud activation via multiple signaling pathways, including trehalose-6-phosphate (T6P) signaling, hexokinase 1 (HXK1)-dependent sugar sensing, and HXK1-driven glycolysis/tricarboxylic acid (TCA) cycle activity and the oxidative pentose phosphate pathway (OPPP). These metabolic pathways support bud growth locally, in part by repressing the central bud dormancy regulator BRANCHED 1 (BRC1). T6P also induces the expression of FLOWERING LOCUS T (FT) and the sugar transporters SWEETs (SUGAR WILL EVENTUALLY BE EXPORTED TRANSPORTERS), which facilitate sugar delivery to developing buds and further reinforce their activation. Such sugar delivery might also be mediated by other transporters, such as SUTs (Sucrose Transporters) and STPs (Sugar Transport Proteins). Conversely, SLs inhibit branching by promoting the degradation of SMXLs repressors via the D14-D3/MAX2-SCF complex, which in turn enhances *BRC1* expression via SPLs (SQUAMOSA PROMOTER BINDING PROTEIN-LIKE). SLs also repress *Trehalose-6-PHOSPHATE SYNTHASE * (*TPS1*), resulting in the reduction of T6P production. Additionally, citrate—a metabolite of the TCA cycle—can interfere with SL perception by reducing the binding of D3/MAX2 to D14 *in vitro*, thereby weakening SL-mediated repression. BRC1/TB1 emerges as a key integrative node modulated antagonistically by sugar availability and SL signaling. SPLs (SPL9 and 15 in Arabidopsis and SPL14 in rice). Blue arrows represent activation, red blunt-ended lines indicate repression.

Shoot branching is a fundamental architectural trait in plants. It influences crop yield, competition for light and adaptation to environmental conditions, including abiotic stress. This developmental outcome depends on the ability of axillary buds to activate and grow or to remain quiescent and dormant. This decision is regulated by a complex interplay of endogenous cues, among which sugar and SL signalings that operate antagonistically [113, 114]. Sugars act as mobile systemic signals that strongly promote bud outgrowth [52, 115]. At least four sugar-related pathways contribute to this regulation: (i) T6P signaling (in Arabidopsis and pea), (ii) HXK1-dependent signaling (in Arabidopsis), (iii) HXK1-dependent glycolytic/TCA cycle (in rose), and (iv) HXK1-dependent OPPP (in rose) (Fig. 4) [20, 32, 116–124]. These pathways emphasize the paramount importance of sugar status perception in determining bud fate. Studies using sugar pathway mutants (e.g. *athxk1*, *tps1* in Arabidopsis) and exogenous effectors (in rose) of sugar metabolism have clearly demonstrated that sugar signaling is tightly correlated with the bud’s capacity to activate and grow [20, 32, 119, 120, 123]. Importantly, these sugar-dependent mechanisms also overlap with hormonal control pathways [118, 125], particularly those involving in SLs. Unlike sugars, SLs inhibit shoot branching by enforcing bud dormancy (Fig. 4). Indeed, SL-defective mutants, including *MAX *genes in Arabidopsis; *ramosus* (*rms*) in pea, *decreased apical meristem* (*dad*) in petunia, and *dwarf* (*d*)/*high tillering dwarf* (*htd*) in rice exhibited enhanced shoot branching [52]. SLs promote the proteasomal degradation of the co-repressors SMXL6/7/8 in Arabidopsis and D53 in rice , which are inhibiting transcription of suppressor of bud outgrowth [62, 126]. SMXLs downregulate *SPL* transcription factors (*SPL9* and *SPL15* in Arabidopsis and *SPL14* in rice), which are positive regulators of *BRC1* (*BRANCHED1*), thereby further reinforcing bud growth suppression (Fig. 4) [127, 128]. Recent findings indicate that SMXL5, which integrates into the SMXL complex, may dampen SL repression by modulating this degradation process [129]. The central node where sugar and SL pathways converge is the TCP transcription factor *BRC1* (*BRANCHED1*) in dicots, or its ortholog *TB1* (*TEOSINTE BRANCHED1*) in monocots (Fig. 4). This transcription factor acts as a master repressor of bud outgrowth [122]. SLs promote *BRC1*/*TB1* expression to maintain dormancy, whereas sugars suppress it to enable bud activation [72, 118, 130]. This sugar-mediated downregulation of *BRC1* occurs independently of the T6P pathway in Arabidopsis and pea, suggesting the existence of alternative, as yet unexplored, sugar-responsive signaling routes [121]. Sugars also negatively regulate key components of SL biosynthesis and signaling, thereby promoting resistance to SL-mediated repression. For example, sugar treatment lowers *MAX2* expression in rose [118] and decreases the rate at which SLs degrade D53 in rice, thereby reducing the SL signal downstream [131] (Fig. 4). Sugar metabolism also intersects with SL signaling through the TCA cycle: citrate, a TCA intermediate, interferes with SL signaling by disrupting D3/MAX2 binding to D14 *in vitro*, thereby impairing SL-mediated repression of bud outgrowth [132] (Fig. 4). However, this mechanism still requires validation *in planta*. SLs can inhibit sugar signaling, especially the T6P pathway. T6P levels rise rapidly (within six hours) following decapitation of the shoot apex, coinciding with increased bud size and first signs of activation [119]. T6P positively regulates both local and systemic bud outgrowth by enhancing sucrose allocation to buds and upregulating FT (*FLOWERING LOCUS T*) [32]. Furthermore, T6P promotes the expression of sugar transporters, including *SWEET11* and *SWEET12 (SUGAR WILL EVENTUALLY BE EXPORTED TRANSPORTERS)*, which are crucial for phloem loading and sugar supply to developing buds, as well as *SWEET13* and *SWEET14*, which are specifically expressed in buds [32]. This reinforces the sucrose–T6P–bud outgrowth axis. SLs antagonize this route by suppressing TPS1 (Trehalose Phosphate Synthase 1), the primary enzyme responsible for T6P synthesis in Arabidopsis and pea [121], thereby hindering the T6P-mediated bud activation program. Taken together, the antagonistic relationship between sugars and SLs orchestrates a complex regulatory network that determines the fate of axillary buds and, consequently, shoot branching. Notably, this antagonism could also extend to other developmental contexts, such as tuber formation in potato. Silencing *CCD8* reduces tuber-bud dormancy, leading to aerial tuber formation from axillary buds [133]. This phenotype correlates with reduced expression of *StBRC1b*, a paralog of *BRC1* that promotes bud dormancy against tuberization [134], highlighting the conserved role of *BRC1-like* factors in mediating SL-sugar interactions across shoot and underground organs. Future research should explore whether SLs modulate other sugar signaling pathways—particularly those involving HXK1, glycolysis, the TCA cycle, and the OPPP—and identify the molecular mediators of sugar–SL crosstalk.

## Sugar–SL crosstalk in leaf senescence: dual sugar roles and hormonal interactions

Leaf senescence is a highly regulated developmental process that facilitates the recycling and remobilization of nutrients to reproductive tissues. It is triggered by developmental cues, carbon status, and environmental factors, and is mediated by the complex interplay of hormonal and metabolic signals [135]. Sugars promote leaf senescence by triggering transcriptional and physiological changes. Two key sugar pathways are involved: HXK1 and T6P. In Arabidopsis, rice and tomato, overexpressing *HXK1* accelerates senescence, while HXK1 catalytically inactive or HXK1-deficient mutants delay it [22, 25]. This emphasizes the significance of glucose sensing, as opposed to merely metabolism, in the regulation of senescence. T6P promotes senescence, partly by inhibiting SnRK1, a known senescence suppressor [136]. Overexpression of T6P synthase (*otsA*) accelerates senescence, whereas overexpression of T6P phosphatase (*otsB*) delays it [137]. Sugar-induced senescence has likely evolved as an adaptive response to optimize carbon remobilization under nutrient-rich or late-season conditions, ensuring reproductive success. SLs promote leaf senescence as SL-biosynthesis and SL-insensitive mutants exhibit a delayed senescence phenotype in Arabidopsis, rice, petunia and *Lotus japonicus* but no altered senescence phenotype has been reported for SL mutants/transgenics in pea or tomato [135]. This SL effect is notably context-specific and SL-induced senescence becomes more pronounced under nutrient deficiencies (e.g. nitrogen and phosphate) and extended darkness [135, 138]. In bamboo and Arabidopsis, SL application enhances chlorophyll degradation, reactive oxygen species (ROS) accumulation and ET-mediated signaling [139]. In such conditions, ET acts synergistically with SLs, and both pathways converge to amplify senescence signals. Evidence suggests that sugars counteract SL-induced senescence. The application of exogenous sugars (such as glucose, fructose and sucrose) delays SL-induced senescence in bamboo leaves exposed to darkness by suppressing the degradation of chlorophyll, accumulation of ROS and the expression of senescence-associated genes (SAGs) [140]. This antagonism may be mediated through the TOR kinase pathway, which responds to high sugar availability by promoting cell survival and repressing senescence. TOR activity reduces ROS levels, maintains chlorophyll content, and inhibits autophagy. Upregulation of TOR by sugar could therefore counteract SL-induced senescence [141]. However, it remains unproven whether TOR acts as a direct integrator of sugar and SL signals during senescence. Deciphering how TOR, SnRK1, and other sugar sensors interface with SL signaling components (e.g. *MAX2*, *SMXLs*) could provide new insights into how plants coordinate senescence with metabolic status and environmental stress.

## When to bloom: sugars and SLs influence the timing of floral transition

**Figure 5:**
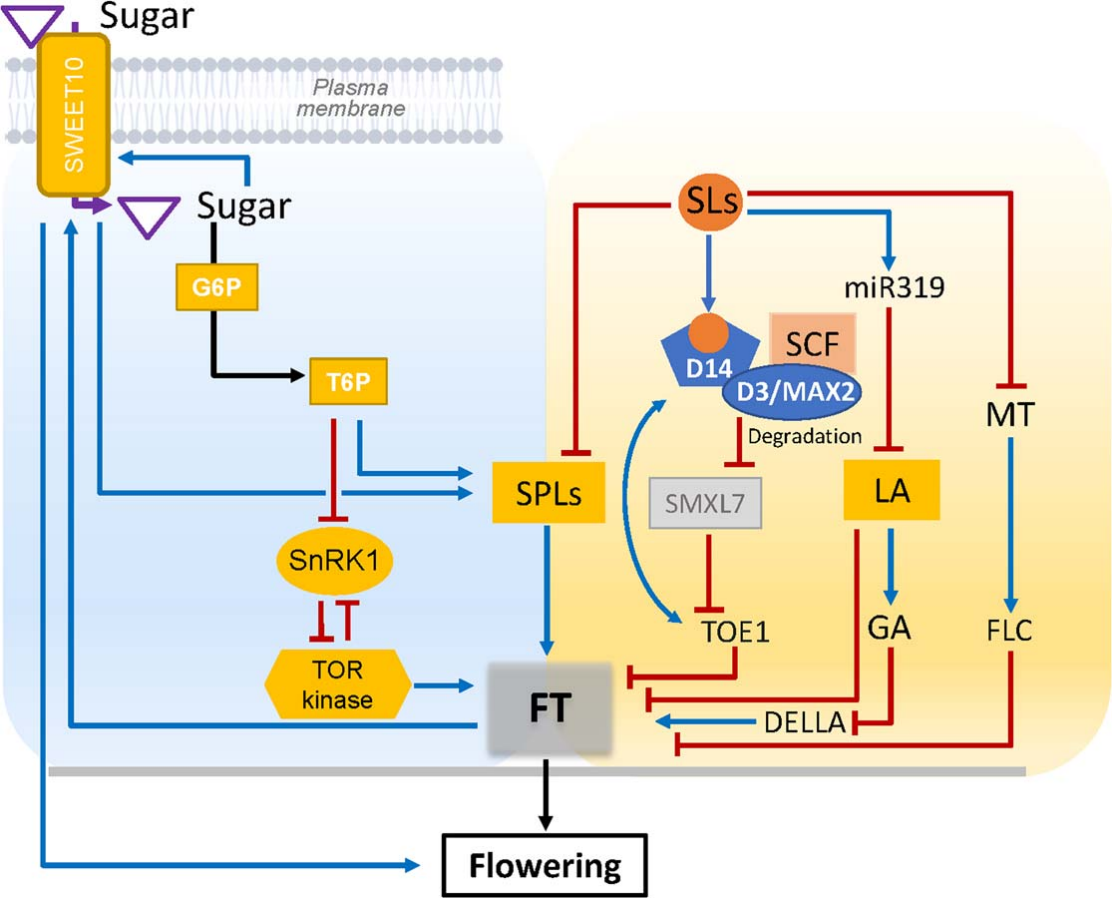
Integration of sugar and SL signaling pathways in the regulation of flowering time. Sugar promotes flowering through multiple routes, including trehalose-6-phosphate (T6P), which represses SnRK1 (Sucrose non fermenting Related Kinase1) and promotes *SPL* (*SQUAMOSA PROMOTER BINDING PROTEIN-LIKE*) gene expression; the induction of the *SUGAR WILL EVENTUALLY BE EXPORTED TRANSPORTER10* (*SWEET10*); and ultimately the activation of the florigen gene FT (*FLOWERING LOCUS T*). SLs regulate flowering through the D14/MAX2 pathway by promoting SMXL7 degradation and stabilizing TARGET OF EARLY ACTIVATION TAGGED 1 (TOE1), a repressor of FT. They also inhibit SPL transcription factors (SPL3, 5, and 9), positive regulators of FT and flowering. In parallel, SLs lower GA levels via the miR319–LANCEOLATE (LA) module, leading to DELLA protein stabilization, which promotes flowering gene expression. Additionally, SLs relieve repression of melatonin-induced *FLC* (*FLOWERING LOCUS C*) upregulation, thereby indirectly enhancing *FT* expression. Although sugar and SL pathways have distinct upstream signals, they converge on shared molecular hubs. Notably, FT, which integrates multiple inductive cues, is positively regulated by both sugar and SL signaling. Blue arrows represent activation, red blunt-ended lines indicate repression.

For horticultural species, the timing and quality of flowering have a direct impact on yield, fruit set and overall market value. This makes regulating them crucial for productivity and commercial success. Flowering is a critical developmental transition regulated by a complex network of genetic and environmental cues. In addition to well-established pathways—such as photoperiod, vernalization, and gibberellin signaling—emerging evidence highlights the importance of metabolic status, particularly sugar signaling and SLs, in modulating flowering time and floral development [142–145]. Sugars function not only as energy sources but also as key floral signals. Three main sugar-related pathways contribute to flowering: T6P and the module TOR kinase and SnRK1 (Fig. 5). First, sucrose acts systemically, especially in leaves, to promote the expression of *FT* (*FLOWERING LOCUS T*), the mobile florigen that initiates flowering at the shoot apical meristem (SAM) [146]. Similarly, sugars (i.e. sucrose) accelerate time flowering in Chrysanthemum by mainly enhancing the level of *CmFTL2* (*Chrysanthemum FT like 2*), compared to *CmFTL1* and *CmFTL3* [147]. In Arabidopsis, FT also enhances sugar export by upregulating *SWEET10*, a sucrose transporter, facilitating increased sucrose flow to the SAM [148]. Ectopic expression of *AtSWEET10* leads to early flowering and elevated expression of flowering-related genes *SPL4* and *SPL9* in the SAM [149]. Second, T6P acts in both leaves and the SAM to reinforce the floral transition downstream of FT [143, 146]. T6P levels increase during floral induction and fine-tune flowering time by regulating *SPL* gene expression, partly through the age-dependent *miR156* pathway, thus linking metabolic cues to developmental timing [143]. TOR and SnRK1 modulate sugar-dependent flowering. TOR promotes flowering under high-sugar conditions by phosphorylating targets such as RPS6 (ribosomal protein S6), thereby enhancing translation and possibly stabilizing FT [149]. In contrast, SnRK1, activated under low-energy conditions, delays flowering by phosphorylating the transcription factor FBH4, which reduces its ability to activate *CONSTANS* (*CO*), a key regulator of *FT* expression [150]. Thus, SnRK1 acts as a repressor of the CO–FT module. Together, these sugar-responsive pathways ensure that flowering is initiated only when energy reserves are adequate to support reproductive growth. SLs also influence flowering time in various species, although their effects are context-dependent (Fig. 5). In tomato, SLs accelerate the floral transition by promoting *miR319*, which inhibits the activity of *LANCEOLATE* (*LA*), a TCP transcription factor in leaves [151]. This repression reduces gibberellin levels, stabilizing the DELLA protein PROCERA, which in turn enhances the expression of *SINGLE FLOWER TRUSS (SFT)*, the tomato ortholog of *FT* [151]. In Arabidopsis, SLs relieve the repression on melatonin-induced *FLC* (*FLOWERING LOCUS C)* expression, which leads to reduced FLC levels and thereby indirectly promotes *FT* expression [152, 153]. Despite promoting flowering, SLs can also repress it. In *Arabidopsis*, SL-deficient and SL-insensitive mutants (e.g. *max3*, *max4*, *d14*, *max2*) flower earlier under both long- and short-day conditions, suggesting that SLs act as repressors of flowering [152]. In this context, SLs suppress *FT* expression, at least in part, by repressing *SPL* genes (*SPL3*, *SPL4*, *SPL9*), which are positive regulators of flowering (Fig. 5). More directly, Bai et al. (2024) [154] showed that the SL receptor D14 interacts with and stabilizes TARGET OF EARLY ACTIVATION TAGGED 1 (TOE1), an AP2-family transcriptional repressor of *FT*. SL perception thus enhances TOE1-mediated repression of *FT*, delaying flowering in Arabidopsis. Moreover, SL-induced degradation of SMXL7, which relieves its inhibitory effect on TOE1, reinforcing this repressive pathway (Fig. 5) [154]. In rice, SLs modulate panicle development and flowering time through a regulatory network involving *D14*, *SPL14* (also known as *IPA1*, Ideal Plant Architecture1), *OsTB1* (the ortholog of *BRC1* in *rice*), and the circadian regulator *OsCCA1*, which integrates both SL and sugar signals [155]. While sugar and SL pathways can act independently to regulate flowering, emerging evidence suggests points of convergence. In citrus, exogenous application of GR24, a synthetic SL analog, increased leaf sucrose and starch content and promoted flowering [156]. In rice, OsCCA1 serves as a convergence point between SL signaling and sugar sensing, influencing tillering and flowering-related gene expression [155]. In summary, *FT* is positively regulated by sugars (via T6P and TOR) and negatively regulated by SLs (notably via repression of *SPL3*, *SPL4*, and *SPL9*) (Fig. 5). These findings highlight the SPL-FT axis as a potential molecular hub where sugar and SL signals converge to regulate flowering time. Whether SLs modulate sugar transport or T6P biosynthesis at the SAM remains an open question.

## Balancing energy and hormonal signals: sugar and SL roles in abiotic stress physiology

**Figure 6:**
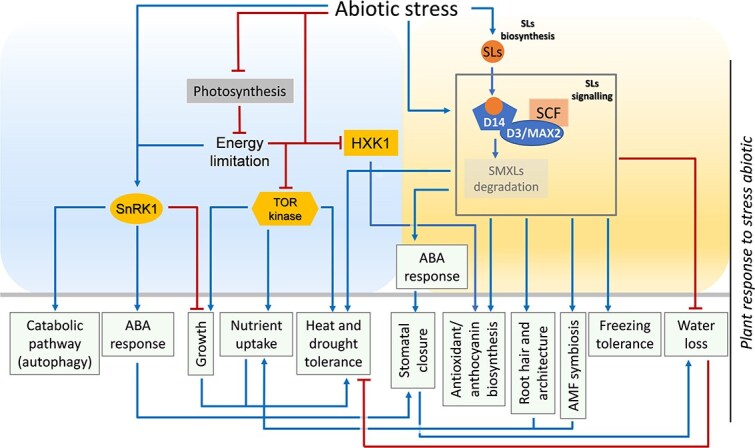
SLs and sugar signaling in abiotic stress responses. Abiotic stresses activate SnRK1 (Sucrose non-fermenting Related Kinase1), which leads to the induction of catabolic pathways, growth inhibition, and activation of the ABA (Abscisic Acid) response, ultimately resulting in stomatal closure. These stresses also suppress TOR (Target Of Rapamycin) kinase activity, negatively affecting growth, nutrient uptake, and stomatal function, thereby contributing to drought and heat tolerance. Similarly, abiotic stresses inhibit HXK1 (Hexokinase1), which also promotes stomatal closure and enhances protection against oxidative stress, notably through the induction of anthocyanin biosynthesis. In parallel, abiotic stresses stimulate SL biosynthesis and signaling, which influence root architecture, promote arbuscular mycorrhizal fungi (AMF) symbiosis and root hair growth, and reduce water loss. These effects collectively improve nutrient uptake and increase tolerance to heat and drought. SLs are also implicated in enhancing freezing tolerance. Finally, SLs and HXK1 converge in the regulation of anthocyanin biosynthesis. Blue arrows represent activation, red blunt-ended lines indicate repression.

Plants are frequently exposed to abiotic stresses such as drought, salinity, nutrient deprivation, extreme temperatures, and oxidative pressure. These stresses are challenging energy homeostasis and threaten survival. Sugar and SL signaling pathways are both crucial for plant adaptation to such stresses in crops [157, 158] and horticultural species [54, 159]. Sugar signaling modulates plant stress responses through central metabolic sensors such as TOR-kinase, SnRK1, and HXK1 [160]. Abiotic stress typically reduces photosynthetic efficiency and carbon assimilation, resulting in lower levels of sucrose, glucose, and T6P [36, 161]. TOR kinase, SnRK1 and HXK1 coordinate metabolic reprogramming to ensure cellular survival (Fig. 6). SnRK1 is activated under energy-deficient conditions such as drought, nutrient limitation, and salinity [14, 46, 162]. Upon activation, SnRK1 represses growth, promotes catabolic pathways including autophagy [163, 164], whilst also enhancing ABA-dependent stress responses [165, 166]. Conversely, TOR kinase is typically inhibited under stress conditions such as heat, salinity, osmotic stress and oxidative stress [167, 168]. However, TOR activation could enhance drought and heat tolerance, increase osmolyte accumulation, and improve nutrient uptake (e.g. potassium) in crops like rice and Arabidopsis [168–171]. TOR also facilitates growth recovery when favorable conditions return [172]. Therefore, maintaining a dynamic balance between SnRK1 and TOR signaling is essential for preserving source–sink relations, carbon partitioning, and cell viability under stressful conditions [173]. HXK1 contributes to stress resilience by regulating stomatal aperture, promoting antioxidant defenses, and inducing anthocyanin biosynthesis, functions that are critical for protection against oxidative and light stress [174, 175]. The interplay between TOR, SnRK1, and HXK1 is often glucose-dependent [14], highlighting a complex sugar-mediated regulatory network that adjusts physiological responses to abiotic stress. SLs enhance tolerance to abiotic stress through various mechanisms involving both the shoot and root systems. In line with this, SLs enhance tomato resilience to cold and heat stress [67], improve salt tolerance [176], and increase drought resistance in apple through a signaling cascade involving MsABI5 (Abscisic Acid Insensitive 5)-MsSMXL1–MsNAC022 [177]. In Arabidopsis, SL-deficient and SL-insensitive mutants (e.g. *max2*) exhibit heightened sensitivity, including accelerated water loss and reduced survival rates under drought and salinity conditions [178] and SLs are reported to promote drought resistance by regulating stomatal closure through crosstalk with ABA [179, 180]. During cold stress, SLs enhance freezing tolerance by promoting the degradation of the transcriptional repressor WRKY41, a member of the WRKY family transcription factor. This enables the activation of CBF (C-repeat binding factor), a cold-responsive gene, via MAX2-mediated ubiquitination [181]. In the root system, SLs modify RSA in order to enhance the uptake of water and nutrients. Depending on nutrient availability, particularly phosphate and nitrogen, they promote root hair development and alter LR formation in Arabidopsis [182] and in tomato [183]. SLs also facilitate the recruitment of arbuscular mycorrhizal fungi (AMF), enhancing the acquisition of phosphorus and water under drought or low-nutrient conditions [10], including in tomato and lettuce under drought [184]. Intriguingly, SLs are produced even in non-AMF-host species, implying that they may directly regulate RSA to optimize adaptation to nutrient limitations [185, 186]. Collectively, these findings position SLs as integrators of environmental signals, enabling adaptive responses that optimize resource use under abiotic stress [12]. Several lines of evidence indicate that sugar and SL signaling pathways converge to enhance stress tolerance. For example, HXK1, trehalose, and SLs jointly regulate ABA signaling, stomatal closure, and the activation of anthocyanin and antioxidant defences [165, 187–189]. Future research should further investigate the molecular mechanisms underlying these integrative hubs, including whether sugar availability modulates SL sensitivity and signaling under stress, and how this interaction influences key developmental and physiological processes.

## Sugars and SLs as dual regulators of plant biotic stress responses

**Figure 7:**
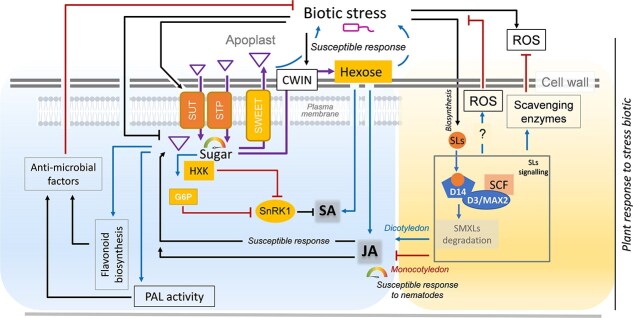
SLs and sugar signaling in biotic stress responses. Sugar localization is a critical factor in determining the host’s susceptibility or resistance to bio-aggressors. The allocation or sequestration of sugars is regulated by Sucrose Uptake Transporters (SUT), Sugar Transporter Proteins (STP), and SUGAR WILL BE EVENTUALLY EXPORTED TRANSPORTER (SWEET) that mediate fluxes between plant cells and the apoplast, as well as by Cell Wall Invertase (CWIN)-dependent sucrose hydrolysis into hexoses. High intracellular sugar levels activate the hexokinase (HXK)-dependent signaling pathway, which positively regulates phenylalanine ammonia-lyase (PAL) activity and flavonoid biosynthesis, leading to the production of antimicrobial compounds. Sugar signaling also lifts the Sucrose non fermenting Related Kinase1 (SnRK1)-dependent repression of the salicylic acid (SA) pathway. Both the SA and jasmonic acid (JA) defense pathways are simultaneously activated by CWIN-mediated hexose accumulation. Moreover, SL biosynthesis is induced by biotic stress. SL signaling, mediated via the D14–MAX2–SCF complex, can modulate the JA pathway in a species-dependent manner—stimulating it in dicotyledons (enhancing resistance) and inhibiting it in monocotyledons (promoting susceptibility). SL signaling is also involved in maintaining reactive oxygen species (ROS) homeostasis and contributing to detoxification mechanisms during plant stress responses. Arrows represent activation, blunt-ended lines indicate repression.

The implication of sugar signaling and localization in defense mechanisms has been described in several species including Arabidopsis, wheat, rice, maize, cotton, barley, sweet potato, tomato, and grapevine [190]. Soluble sugars—sucrose, glucose, and fructose—are primary energy sources that also function as signaling molecules, critically modulating plant defense pathways [191–195]. During plant-pathogen interactions, the localization and concentration of sugars within host tissues significantly influence disease outcomes [193]. Pathogens often exploit host-derived sugars for growth, while plants restrict sugar availability to reinforce defense—a process requiring tight metabolic reprogramming [191, 196]. Pathogens can hijack sugar efflux transporters, particularly those belonging to the *SWEET* family (Fig. 7). The SWEET family plays key roles in sugar efflux and accumulation, impacting fruit development and pathogen interactions in grapevine and in apples. In rice, Xanthomonas species use transcription activator-like (TAL) effectors to activate *SWEET* genes (e.g. *OsSWEET11*/*13*/*14*), promoting sugar leakage into the apoplast where pathogens initially reside [197–199]. Clubroot diseases, one of the most devastating diseases of horticultural crops, are caused by the obligate biotrophic pathogen *Plasmodiophora brassicae*. It provides a striking case study of host sugar metabolism reprogramming to establish a strong nutrient sink within the developing galls [200, 201]. Upon infection, the pathogen triggers the active translocation of sugars from source leaves to the infected roots, leading to a significant accumulation of glucose and fructose in the galls of susceptible hosts [200]. To achieve this, *P. brassicae* hijacks the host’s sugar transport machinery, particularly members of the SWEET transporter family [200, 201]. In Arabidopsis, infection triggers phloem-specific upregulation of *SWEET11* and *SWEET12*, facilitating sugar delivery to the pathogen [201, 202]. Consistently, Arabidopsis *sweet11* and *sweet12* mutants exhibit delayed disease progression, reduced gall formation, and impaired sucrose transport to the pathogen. Similarly, in the *Brassica rapa–P. brassicae* pathosystem, several *BrSWEET* genes, notably *BrSWEET1a*, *BrSWEET11a*, and *BrSWEET12a*, are strongly upregulated in infected roots, and their silencing reduces disease severity [200, 202]. Collectively, these findings demonstrate how *P. brassicae* co-opts host sugar transporters to secure its nutritional needs and promote disease development. Conversely, sugar influx transporters like STPs (Sugar Transporter Proteins) and SUTs (Sucrose Uptake Transporters) reclaim apoplastic sugars, depriving pathogens of nutrients and supporting resistance [198, 199]. However, STPs may enhance susceptibility in biotrophic interactions by supplying sugars to the extrahaustorial matrix [203]. Pathogens also secrete invertases to hydrolyze sucrose into hexoses, which they import via hexose transporters. Host responses include increased cell wall invertase (CWIN) activity, leading to hexose accumulation and defense activation via SA/JA signaling (Fig. 7) [204–206]. CWINs are essential in defense against hemibiotrophic pathogens in tomato, enhancing resistance through hexose signaling [193]. Sugar accumulation also promotes flavonoid synthesis and Phenylalanine ammonia lyase (PAL) activity, both of which are linked to antimicrobial defense [191]. Some pathogens suppress host CWIN to subvert these defenses [207]. While CWIN is well understood, roles of vacuolar (VIN), cytosolic (CIN) invertases, and sucrose synthase (Sus) remain unclear [208]. HXK1 also promotes defense gene expression [17, 195]. The product of HXK1, G6P, enhances SA-dependent immunity, likely by suppressing SnRK1 [195, 209]. Under sugar-sufficient conditions, elevated G6P levels and reduced SnRK1 activity may boost defense readiness via suppression of PP2C phosphatases, an essential factor in ABA signaling, and activation of SA biosynthesis pathways [210]. SLs also significantly enhance horticultural plant defense against pathogens and pests. SLs modulate plant responses to biotic stresses, although their roles can differ depending on the plant-pathogen system (Fig. 7) [211, 212]. SLs mediate interactions with parasitic plants and nematodes via the D14-MAX2 signaling pathway, often acting as susceptibility factors [213, 214]. In monocots like rice, SL-deficient mutants exhibit reduced infection by root-knot nematodes, indicating that SLs suppress JA signaling and promote susceptibility by favoring sugar accumulation (Fig. 7) [215]. Conversely, in dicots like tomato and Arabidopsis, SLs generally enhance resistance to pathogens such as *Botrytis cinerea*, *Alternaria alternata*, *Pseudomonas syringae*, and *Rhodococcus fascians* by promoting SA/JA/ABA-mediated responses [212, 216]. Although SLs are generally implicated in plant defense against various phytopathogenic fungi [217], often by inhibiting their growth, their specific role in clubroot diseases remains largely unexplored. SLs also influence responses to oxidative stress by upregulating the activity of antioxidant enzymes and modulating the ROS-associated gene expression [24, 214]. In grapevine and tobacco**,** GR24 reduces *Botrytis cinerea* infection by increasing fungal ROS [218, 219]. SLs mediate stomatal closure, a key innate immune response that prevents pathogen entry; Arabidopsis SL-deficient mutants show impaired closure and increased susceptibility to *Pectobacterium carotovorum*, which infects crops like cabbage, onion, carrot, and potato [217]. Current evidence suggests that indirect interactions occur through shared hormone networks, notably JA and ABA [192, 213]. For instance, in rice, SLs may suppress JA responses and promote sugar accumulation, thereby increasing susceptibility to *Meloidogyne graminicola* [215]. Similar SL-sugar-hormone interactions have been observed in tomato and tobacco [211, 220]. Furthermore, SLs may modulate sugar metabolism and transport during infection. In *R. fascians*-induced leafy gall syndrome, pathogen-induced SL biosynthesis counteracts the developmental changes mediated by CKs, possibly intersecting with sugar signaling [196]. However, the exact mechanisms remain unclear. These findings highlight the pivotal roles of sugars and SLs in regulating plant immunity, through metabolic functions, signal transduction pathways, and interactions with other phytohormones. Components of sugar signaling such as HXK1 and SnRK1, as well as SL signaling such as the D14–MAX2 module, independently modulate plant–pathogen interactions. However, further investigation is needed to establish whether they interact through modulation of hormonal networks, including JA, ABA, and SA.

## Conclusion and perspective

It is crucial to understand how plants balance growth and resilience in response to fluctuating environmental conditions, particularly in horticultural species where productivity, stress tolerance and developmental plasticity are closely linked. This review emphasizes the pivotal function of sugars and SLs as dual regulators that integrate metabolic status and hormonal cues to modulate vital developmental processes and stress responses. While sugars generally promote anabolic growth, shoot branching and flowering, SLs often act as antagonistic signals that enforce developmental restraint, such as maintaining bud dormancy, reducing LR formation and accelerating senescence, in order to preserve resources under suboptimal conditions. The interplay between these two pathways emerges as a dynamic regulatory module that fine-tunes developmental outcomes based on environmental and internal cues. Integration nodes such as BRC1/TB1 in shoot branching, FT/SPLs in flowering, SMXL proteins in signaling and the TOR–SnRK1–T6P triad in energy sensing are compelling targets for further investigation. For instance, SMXL proteins, key repressors of SL signaling, are destabilized by SLs and stabilized by sugars in the context of shoot branching. However, whether their stability is directly controlled by sugar metabolic or signaling pathways and whether this regulation extends to other sugar-SL controlled processes remains unknown. Additionally, given that both sugars and SLs are involved in modulating hormonal networks - including ABA, JA, SA - understanding how their interplay shapes plant resilience and immunity is a critical research priority. Importantly, emerging data suggest that sugar availability can modulate SL biosynthesis, sensitivity, and downstream repression, while SLs may provide feedback on sugar signaling and transport, particularly through modulation of trehalose metabolism (including T6P) and sucrose allocation. This antagonistic yet coordinated regulation is particularly relevant for horticultural species, which often experience episodic stresses and require precise control over architecture and reproductive timing to meet quality and yield standards. Overall, incorporating sugar–SL crosstalk into crop improvement strategies could lead to the development of context-responsive plants that balance growth vigor with adaptive restraint. This is particularly critical in the face of climate variability and the growing need for sustainable, high-performance crop systems.
